# Topographic Analysis of Maxillary Posterior Teeth and Maxillary Sinus in the Mongolian Population

**DOI:** 10.7759/cureus.84560

**Published:** 2025-05-21

**Authors:** Namuunzul Yondon, Urangua Erdenechuluun, Khatanzaya Ulziisaikhan, Nominzaya Munkhtur, Enkh-Orchlon Batbayar, Oyuntugs Rashsuren, Delgertsetseg Jargaltsogt

**Affiliations:** 1 Dentistry, School of Dentistry, Mongolian National University of Medical Sciences, Ulaanbaatar, MNG; 2 Oral and Maxillofacial Surgery, School of Dentistry, Mongolian National University of Medical Sciences, Ulaanbaatar, MNG; 3 Oral and Maxillofacial Radiology, Central Dental Hospital, Mongolian National University of Medical Sciences, Ulaanbaatar, MNG; 4 Dental Hygiene, School of Dentistry, Mongolian National University of Medical Sciences, Ulaanbaatar, MNG

**Keywords:** alveolar bone, cone-beam computed tomography, molars, premolars, sinus floor

## Abstract

Background and objectives: The relationship between the maxillary posterior teeth and the level of the maxillary sinus varies across different populations and can significantly impact the success of endodontic, prosthodontic, orthodontic, and surgical treatments. This study aimed to evaluate the alveolar bone anatomy of maxillary posterior teeth and the vertical relationship to the maxillary sinus in Mongolian adults using cone-beam computed tomography (CBCT) images.

Materials and methods: The study was designed as a retrospective study. We selected 30 CBCT images and examined a total of 202 maxillary posterior teeth using these CBCT images. We measured the alveolar bone thickness at six different distances (L1-L6) by millimeter scale, and the vertical relationship between the maxillary sinus and the maxillary molars was classified into five categories according to Kwak's classification. Statistical analyses were done using IBM SPSS Statistics for Windows, Version 27 (Released 2020; IBM Corp., Armonk, New York).

Results: The average distances measured were 1.66±1.10 mm at L1, 2.13±1.00 mm at L2, 3.03±2.21 mm at L3, 6.04±3.50 mm at L4, 2.41±3.54 mm at L5, and 0.74±2.09 mm at L6, with statistical significance (p<0.001). According to Kwak's classification, the most commonly observed types are Type I (82.1%) at the maxillary first premolars, Type III (63.3%) at the maxillary second premolars, Type II (43.3%) at the maxillary first molars, and Type I (43.3%) at the maxillary second molars (p<0.001).

Conclusion: The horizontal distances between the buccal and palatal roots to the alveolar plate were shorter in molars than in premolars due to root numbers, indicating a narrower bone structure that may limit implant stability. Additionally, the vertical distance between the root apex and the maxillary sinus was shortest in second molars, increasing the risk of sinus perforation during implant placement.

## Introduction

A dental implant is considered one of the treatment options for complete and partial edentulism [1]. The success rate for dental implants ranges from 88% to 92% [2]. Mohammadi et al. reported that the success rate of maxillary implants is lower than that of mandibular implants [3]. Post-extraction resorption in the maxillary molar and premolar regions and the associated sinus pneumatization have only been recently investigated qualitatively and quantitatively using 3D imaging techniques [4,5]. The systematic review and meta-analysis concluded that the prevalence of postoperative infection or sinusitis after implant surgery is low, and it may depend either on the dimensions of the perforation or the anatomical predisposition [6]. The majority of dental clinicians use cone-beam computed tomography (CBCT) in the diagnosis, assessment, planning, and delivery of treatment in various specialties in dentistry. The most common indication was evaluating bone and teeth for implant planning in the maxilla [7].

Chen et al. observed that edentulism continues to be a significant global public health concern, particularly as its prevalence is expected to rise with the aging population [8]. Edentulism, or tooth loss, has been examined in relation to multiple health-related behaviors [9] and considerably affects oral health-related quality of life (OHRQoL), causing chewing difficulty, poor dietary intake, and functional disorders [10]. Most Mongolian elderly people did not have their own natural teeth [11]. Most patients prefer to use fixed dentures rather than removable dentures [12]. Karthik et al. described that the basic criteria for implant success are immobility, absence of peri-implant radiolucency, adequate width of the attached gingiva, and absence of infection [13]. The edentulous posterior maxilla is considered a clinical challenge during dental implant treatment due to the high risk of maxillary sinus septa perforation. Therefore, it can be suggested that preoperative radiographic evaluation is helpful for diagnosis and treatment planning and minimizing complications during the surgery [14].

In Mongolia, the number of dental clinics using CBCT is increasing significantly, and most implant patients now undergo CBCT scanning prior to treatment. However, few CBCT-based topographic studies have specifically investigated alveolar bone width and height and the relationship between maxillary molars and the maxillary sinus in Mongolia [15,16].

There have been insufficient studies evaluating the alveolar bone morphology of the posterior maxilla and its vertical relationship to the maxillary sinus, specifically among Mongolian adults. This study aimed to evaluate the alveolar bone anatomy of maxillary posterior teeth and the vertical relationship to the maxillary sinus in Mongolian adults using CBCT images.

## Materials and methods

The study design was a retrospective study. We selected CBCT images based on the following inclusion criteria: visible maxillary posterior teeth and maxillary sinus floor, absence of traumatic injuries, and no presence of orthodontic and orthopedic appliances. All images were taken in the Department of Oral and Maxillofacial Radiology, Central Dental Hospital, Mongolian National University Medical Sciences (MNUMS), between 2021 and 2024. We used full CBCT (16 cm × 8 cm) images, which were obtained with HDX, WILL (DENTRI, Seoul, Korea) (85 kW, 7 mA) using OnDemand3D (CyberMed, Seoul, Korea). The area of slice thickness on CBCT was 0.125, and the rotation time was 17.5 seconds. A total of 202 maxillary posterior teeth were examined using 30 CBCT images. To eliminate operator error, the two examiners performing CBCT images measured and marked the cross-sectional plane; in 10 randomly selected cases, all measurements were made twice to assess intra-rater reliability at two-week intervals using Cronbach's alpha (α = 0.98).

**Figure 1:**
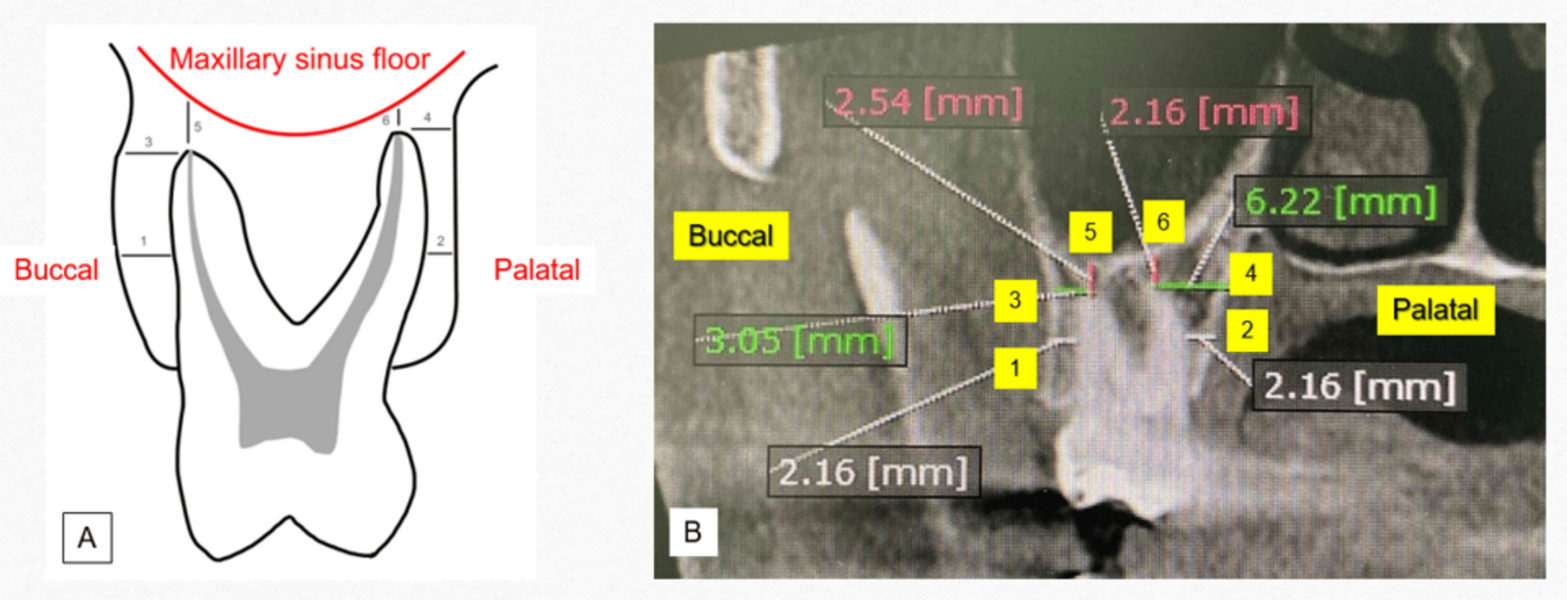
Linear measurements between the maxillary premolars and molars and the adjacent anatomical structures in the plane of CBCT images. (A) Schematic illustration of parameters used for linear measurements; (B) actual linear measurements on the coronal view of the CBCT scan. (1) Horizontal distance between the midpoint of the buccal root and the buccal alveolar plate (L1); (2) horizontal distance between the midpoint of the palatal root and the palatal alveolar plate (L2); (3) horizontal distance between the buccal root apex and the buccal alveolar plate (L3); (4) horizontal distance between the palatal root apex and the palatal alveolar plate (L4); (5) vertical distance between the buccal root apex and the inferior wall of the maxillary sinus (L5); (6) vertical distance between the palatal root apex and the inferior wall of the maxillary sinus (L6) Image Credits: Namuunzul Yondon

We measured alveolar bone thickness using the six linear measurements (four horizontal, L1-L4, and two vertical, L5-L6) at the maxillary posterior teeth on the cross-sectional plane of CBCT images using OnDemand3D software (Figure 1) [17].

**Figure 2:**
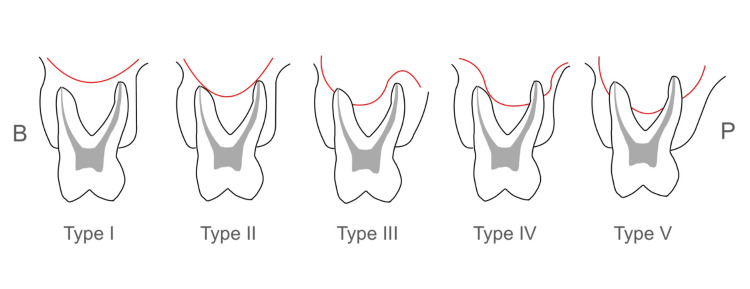
Classification of the vertical relationship between the maxillary sinus and the roots of the maxillary premolars and molars, in accordance with Kwak et al. Type I: The inferior wall of the maxillary sinus was located above the level connecting the buccal and palatal root apices. Type II: the inferior wall of the sinus was located below the level connecting the buccal and palatal root apices, without an apical protrusion over the inferior wall of the maxillary sinus. Type III: an apical protrusion of the buccal root apex was observed over the inferior wall of the maxillary sinus. Type IV: an apical protrusion of the palatal root apex was observed over the inferior wall of the maxillary sinus. Type V: an apical protrusion of the buccal and palatal root apices was observed over the inferior wall of the maxillary sinus B: buccal side; P: palatal side Image Credits: Namuunzul Yondon

We classified the vertical relationship between the inferior wall of the maxillary sinus and the maxillary posterior teeth into five types according to Kwak et al.'s classification (Figure 2) [17].

Statistical analysis

All data were evaluated using IBM SPSS Statistics for Windows, Version 27 (Released 2020; IBM Corp., Armonk, New York). After checking for outliers in each measurement level (x ≤ Q1 - 1.5IQR; x ≥ Q3 + 1.5IQR), the Kolmogorov-Smirnov test was used to assess the normal distribution. If the p-value was greater than 0.05, it was considered to follow a normal distribution. To calculate the differences in the linear measurements of height and width of the alveolar bone on the maxillary posterior teeth, a one-way ANOVA test was used. If the one-way ANOVA test showed significant differences, the Tukey test was applied to compare the pairs of measurements. If the p-value was less than 0.05, the difference was considered statistically significant.

## Results

A total of 202 maxillary posterior teeth were examined using 30 CBCT images of the patients, whose average age was 26.87 years (range, 16-42 years).

Measurement of the alveolar bone thickness by six linear measurements

We evaluated the four horizontal, L1-L4, and two vertical, L5-L6, distances at the maxillary posterior teeth on the cross-sectional plane of the CBCT images, which are shown in Table 1. The mean distances of L1-L6 were 1.66±1.10 mm, 2.13±1.00 mm, 3.03±2.21 mm, 6.04±3.50 mm, 2.41±3.54 mm, and 0.74±2.09 mm, respectively, in all maxillary posterior teeth, which were statistically significant. The shortest distance of L1 was 1.08±0.55 mm in the first premolar, of L2 was 1.42±0.83 mm in the first molar, of L3 was 1.51±1.11 mm in the first premolar, of L4 was 3.30±2.38 mm in the first molar, of L5 was 0.467±1.37 mm in the first molar, and of L6 was 0.69±2.42 mm in the first premolar, significantly. The thickest alveolar bone of all six linear measurements was 8.45±2.42 at the L4 distance of the maxillary second premolar (Table 1). The vertical distance between the buccal root apex and the inferior wall of the maxillary sinus (L5) was maximal at the maxillary first premolars (5.14±4.32 mm) and minimal at the maxillary second molars (0.67±1.36 mm) (p<0.001). Additionally, the vertical distance between the palatal root apex and the inferior wall of the maxillary sinus (L6) was maximal at the maxillary first molars (1.25±2.53 mm) and minimal at the maxillary first premolars (0.69±2.42 mm), which is significant.

**Table 1:** Linear measurements of the horizontal and vertical distances between the sides of the alveolar bone at the maxillary posterior teeth on the cross-sectional plane of CBCT images (mm). Results of the one-way ANOVA

Tooth	N	Distance	L1	L2	L3	L4	L5	L6
First premolar	53	Mean±SD	1.08±0.55	2.81±0.92	1.51±1.11	8.02±2.80	5.14±4.32	0.69±2.42
		Min-max	0.01–3.45	0.83–4.85	0–4.43	1.36–14.45	0.0–16.47	0.0–13.39
Second premolar	58	Mean±SD	1.68±0.93	2.38±0.80	2.97±1.66	8.45±2.42	2.20±3.17	-
		Min-max	0.52–7.01	1.21–5.34	0.02–9.42	4.35–13.97	0.0–11.06	-
First molar	45	Mean±SD	1.11±0.67	1.42±0.83	2.72±1.44	3.30±2.38	1.25±2.63	1.25±2.53
		Min-max	0.01–3.5	0.32–4.18	0.45–6.52	0.5213.61	0.0–13.63	0.0–9.63
Second molar	46	Mean±SD	2.83±1.19	1.72±0.86	5.16±2.74	3.38±2.40	0.67±1.36	1.24±2.28
		Min-max	0.61–5.74	0.67–4.3	0–12.39	1.09–11.54	0.0–6.4	0.0–10.82
Total	202	Mean±SD	1.66±1.10	2.13±1.00	3.03±2.21	6.04±3.50	2.41±3.54	0.74±2.09
p-value			p<0.001	p<0.001	p<0.001	p<0.001	p<0.001	p<0.01

**Figure 3:**
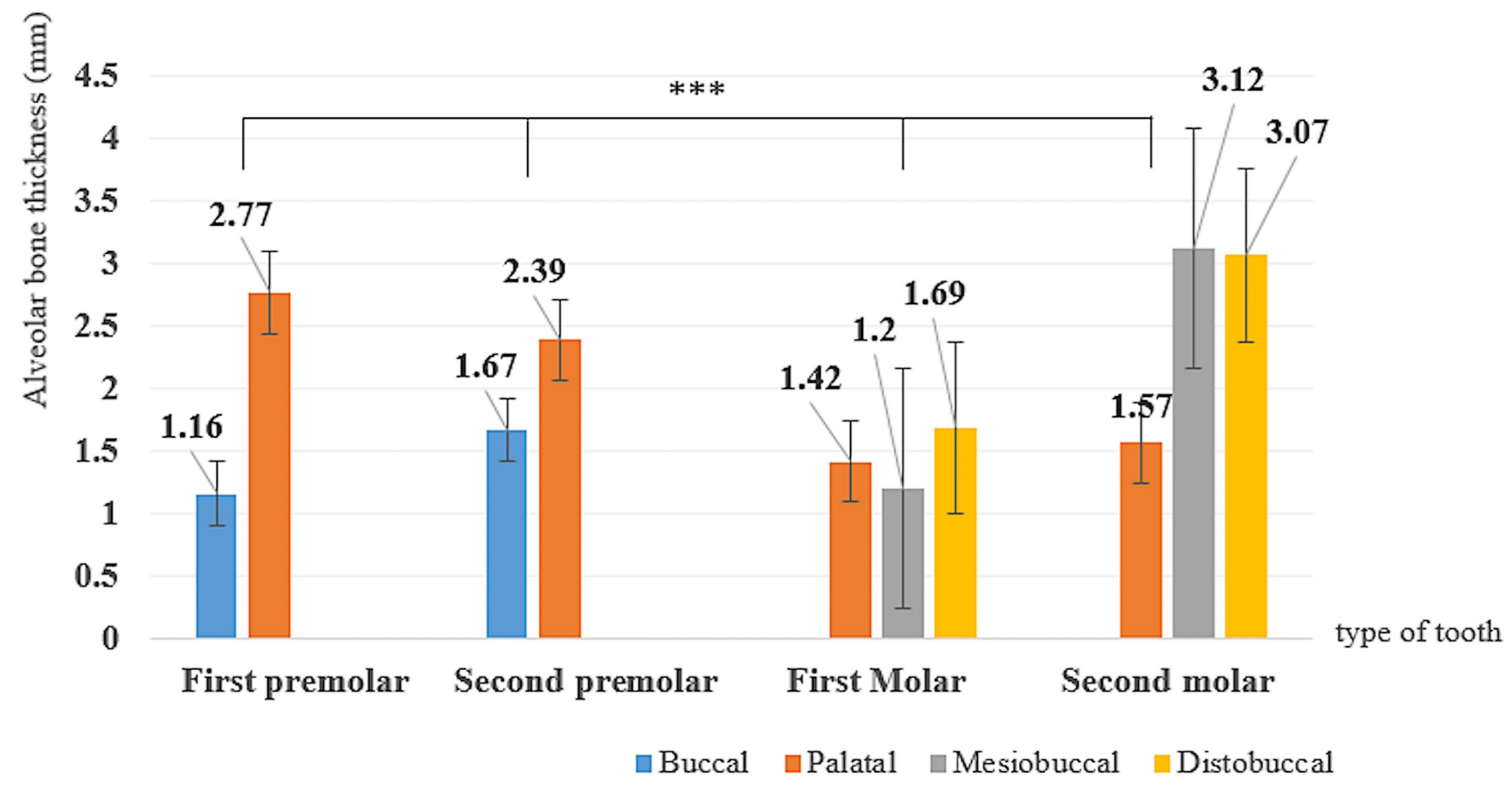
Bar graph showing the average horizontal distance (L1 and L2 measurement) between the midpoint of the root and the alveolar plate for each tooth (mean±SE). *** p<0.001. Results of the one-way ANOVA SE: standard error

Figure 3 illustrates the measurement of distances from the midpoints of the root to both the buccal (L1) and palatal (L2) alveolar plates.

The buccal alveolar bone thickness of premolars was significantly smaller than that of molars, and the palatal was significantly bigger. We observed that the shortest L1 distance in the maxillary molar region was at the mesiobuccal root of the first molar, and the longest was at the mesiobuccal root of the second molar (p<0.001) (Figure 3).

**Figure 4:**
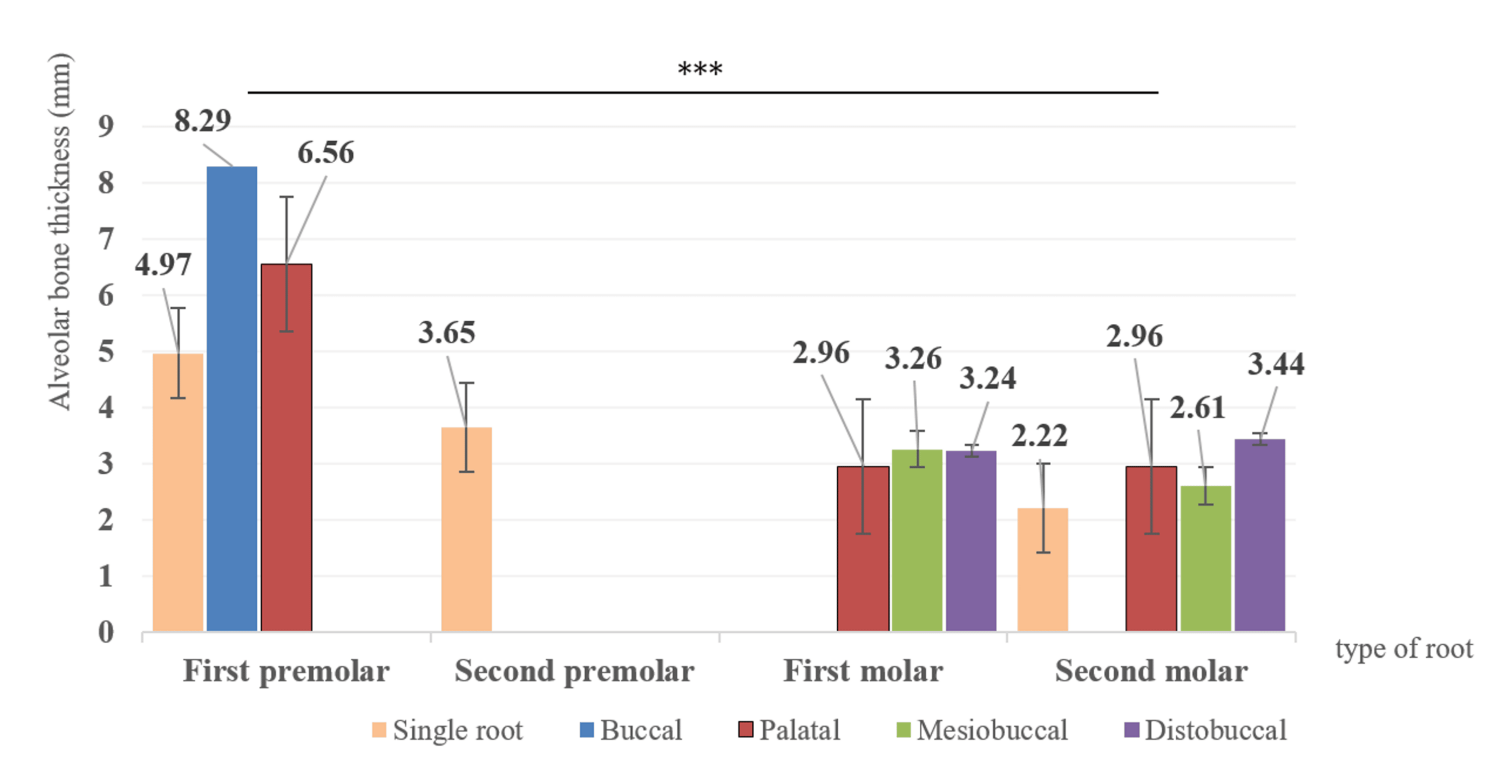
Bar graph showing the average vertical distance (L5 and L6 measurement) between the apices of the root and the maxillary sinus floor for each tooth (mean±SE). *** p<0.001. Results of the one-way ANOVA SE: standard error

We measured the distances between the root apices of the maxillary posterior teeth and the inferior wall of the sinus of the Mongolian adults. Figure 4 shows that the average value of the maxillary first premolar's buccal root was the greatest distance (8.29 mm), and the maxillary second molar, which had a single root, was the shortest distance (2.22 mm), which was statistically significant (p<0.001). The first and second molars are located nearer to the maxillary sinus floor than the premolars among Mongolians (Figure 4).

Vertical distributions between the maxillary sinus floor and the maxillary posterior teeth

**Figure 5:**
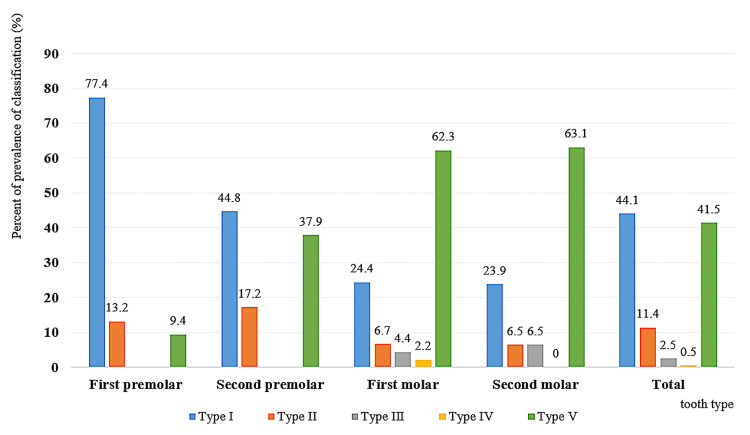
Bar graph showing the distribution of the vertical relationship classification between the maxillary sinus and the roots of the maxillary premolars and molars according to Kwak et al. Results of the one-way ANOVA

We classified the distributions of vertical relationships between the maxillary sinus floor and the maxillary posterior teeth on the cross-sectional plane of CBCT images into five categories, according to Kwak et al.'s classification (Figure 5).

Among the 202 evaluated teeth, 89 (44.1%) were classified as Type I, 23 (11.4%) as Type II, five (2.5%) as Type III, one (0.5%) as Type IV, and 84 (41.5%) as Type V. For the maxillary first premolars, Type I was observed in 41 of 53 cases (77.4%); Type II was in seven cases (13.2%); Type V was in five cases (9.4%); and Types III and IV were absent. Types III and IV were also absent at the maxillary second premolars in Mongolians, and the most commonly observed types, I (44.8%) and V (37.9%), of all second premolars were significantly different. Type V was observed most commonly at the maxillary first and second molars in 62.3-63.1%. Type I was observed in 11 of 45 cases (24.4%) among all maxillary first molars and 11 of 46 cases (23.9%) among all maxillary second molars.

## Discussion

This study aimed to evaluate the morphological characteristics of maxillary molars using CBCT images and to provide clinicians with valuable information for endodontic, periodontal, or orthodontic treatment [18]. The results of this study confirmed that alveolar bone morphology and the vertical relationship between the maxillary posterior teeth and the maxillary sinus vary depending on tooth position. This study evaluated four horizontal distances between the maxillary posterior teeth and the alveolar bone plates, two vertical distances between the root tips and the maxillary sinus floor, and the vertical relationship between the maxillary posterior teeth and the maxillary sinus. Data from 30 CBCT images of Mongolian adults showed that first premolars are located at the greatest distance from the sinus floor, whereas second molars are in closest proximity.

In Mongolia, the inter-root distance and cortical bone thickness of the maxillary teeth were measured using CBCT images [15,16], which is useful for the placement of mini-implants during orthodontic treatment. Heimes et al. [19], López-Jarana et al. [20], and Shafizadeh et al. [21] have concluded that buccal alveolar bone thickness is generally thicker in the molar region compared to the premolar region. However, our study results have identified the following sequence of buccal thickness: the second molar, the second premolar, the first molar, and the first premolar. In the study by López-Jarana et al. [22], the palatal alveolar bone thickness in the maxillary premolar area is generally thinner than in the molar area; however, our study found the opposite. This difference may depend on the vertical level of the horizontal measurement.

When we measured the thickness between the root apex of the maxillary posterior teeth and the maxillary sinus floor, the closest to the maxillary sinus was the root apex of single-rooted second molars, and the furthest was the buccal root of the first premolars among maxillary premolars and molars (p<0.01). Estrela et al. reported that the second molar is closer to the maxillary sinus floor than the first molar [23], which was similar to the results of our study. When we assessed the distribution of the vertical relationship classification between the maxillary sinus and the roots of the maxillary premolars and molars, according to Kwak et al., there was a dominant distribution of Types I and V. In another study, the authors concluded that Type II was most commonly seen in the first and second molars, and Type I was often observed in the premolars [24]. Distinctive genetic and anatomic characteristics exist in different populations. Our study investigated and showcased valuable data from Mongolian adults, specifically focusing on the anatomical variations of the alveolar bone in the maxillary posterior teeth.

Knowledge of this study's measurements can guide Mongolian dentists in preparing an optimal treatment plan and help avoid complications during dental surgical, periodontal, endodontic, orthodontic, and prosthodontic procedures, as well as otorhinolaryngologists. The clinical implications suggest that careful evaluation of tooth-specific bone dimensions is essential for surgical planning, particularly in implant placement or sinus augmentation procedures. A limitation of this study is that we were unable to achieve a balanced distribution of CBCT images among participants by age, sex, living region, and ethnic group in Mongolia.

## Conclusions

The horizontal and vertical measurement distances of the maxillary posterior teeth among Mongolian adults were different by tooth location. These findings emphasize the importance of careful preoperative planning using CBCT imaging to select appropriate implant size and position, ultimately improving long-term success and minimizing complications. The maxillary sinus floor is positioned at a lower level in our study population, which may be attributed to a larger maxillary sinus volume.
